# Supplementary material for: TGF-β uses a novel mode of receptor activation to phosphorylate SMAD1/5 and induce epithelial-to-mesenchymal transition
Source: eLife. 2018 Jan 29;7:e31756. doi: 10.7554/eLife.31756 (PMC5832415; doi:10.7554/eLife.31756)
Supplement: Supplementary file 1. [file elife-31756-supp1.docx]

cgccattctgcctggggacgtcggagcaagcttgatttaggtgacactatagaatacaagctacttgttctttttgcaGG

ATCTatggggagtagcaagagcaagcctaaggaccccagccagcgcggtggaggaggttctggaggcggtggaagtggtg

gcggacacaaccgcactgtcattcaccatcgagtgccaaatgaagaggacccttcattagatcgcccttttatttcagag

ggtactacgttgaaagacttaatttatgatatgacaacgtcaggttctggctcaggtttaccattgcttgttcagagaac

aattgcgagagatattgtgttacaagaaagcattggcaaaggtcgatttggagaagtttggagaggaaagtggcggggag

aagaagttgctgttaagatattctcctctagagaagaacgttcgtggttccgtgaggcagagatttatcaaactgtaatg

ttacgtcatgaaaacatcctgggatttatagcagcagacaataaagacaatggtacttggactcagctctggttggtgtc

agattatcatgagcatggatctctttttgattacttaaacagatacacagttactgtggaaggaatgataaaacttgctc

tgtccacggcgagcggtcttgcccatcttcacatggagattgttggtacccaaggaaagccagccattgctcatagagat

ttgaaatcaaagaatatcttggtaaagaagaatggaacttgctgtattgcagacttaggactggcagtaagacatgattc

agccacagataccattgatattgctccaaaccacagagtgggaacaaaaaggtacatggcccctgaagttctcgatgatt

ccataaatatgaaacattttgaatccttcaaacgtgctgacatctatgcaatgggcttagtattctgggaaattgctcga

cgatgttccattggtggaattcatgaagattaccaactgccttattatgatcttgtaccttctgacccatcagttgaaga

aatgagaaaagttgtttgtgaacagaagttaaggccaaatatcccaaacagatggcagagctgtgaagccttgagagtaa

tggctaaaattatgagagaatgttggtatgccaatggagcagctaggcttacagcattgcggattaagaaaacattatcg

caactcagtcaacaggaaggcatcaaaatgggtggaggaggttctggaggcggtggaagtggtggcggaggtagccctga

ctacagtctcgtgaaggctctgcaaatggcacaacagaattttgtcattacagacgcctccctcccagacaaccctatcg

tctacgccagtagagggtttctgacactgacaggctattctctcgaccagatcctgggcaggaactgcaggtttctgcaa

gggccagaaacagacccaagagctgtggataagatcaggaatgccatcaccaaaggcgttgataccagtgtctgtctgct

gaattatagacaggatggcacaaccttctggaatctcttcttcgtggctggactcagagattctaagggcaatattgtca

actacgtcggagtgcagtcaaaggtgagcgaagattatgccaagctgctggtcaacgagcagaacattgagtacaaaggt

gtgcgcaccagtaacatgctgcgcagaaagcccgggtctagttatccgtacgacgtaccagactacgcataaCTCGAGcc

tctagaactatagtgagtcgtattacgtagatccagacatgataagatacattgatgagtttggacaaaccacaactaga

atgcagtgaaaaaaatgctttatttgtgaaatttgtgatgctattgctttatttgtaaccattataagctgcaataaaca

agttaacaacaacaattgcattcattttatgtttcaggttcagggggaggtgtgggaggttttttaattcgcggccgcgg

cgccaatgcattgggcccggtacccagcttttgttccctttagtgagggttaattgcgcgcttggcgtaatcatggtcat

agctgtttcctgtgtgaaattgttatccgctcacaattccacacaacatacgagccggaagcataaagtgtaaagcctgg

ggtgcctaatgagtgagctaactcacattaattgcgttgcgctcactgcccgctttccagtcgggaaacctgtcgtgcca

gctgcattaatgaatcggccaacgcgcggggagaggcggtttgcgtattgggcgctcttccgcttcctcgctcactgact

cgctgcgctcggtcgttcggctgcggcgagcggtatcagctcactcaaaggcggtaatacggttatccacagaatcaggg

gataacgcaggaaagaacatgtgagcaaaaggccagcaaaaggccaggaaccgtaaaaaggccgcgttgctggcgttttt

ccataggctccgcccccctgacgagcatcacaaaaatcgacgctcaagtcagaggtggcgaaacccgacaggactataaa

gataccaggcgtttccccctggaagctccctcgtgcgctctcctgttccgaccctgccgcttaccggatacctgtccgcc

tttctcccttcgggaagcgtggcgctttctcatagctcacgctgtaggtatctcagttcggtgtaggtcgttcgctccaa

gctgggctgtgtgcacgaaccccccgttcagcccgaccgctgcgccttatccggtaactatcgtcttgagtccaacccgg

taagacacgacttatcgccactggcagcagccactggtaacaggattagcagagcgaggtatgtaggcggtgctacagag

ttcttgaagtggtggcctaactacggctacactagaaggacagtatttggtatctgcgctctgctgaagccagttacctt

cggaaaaagagttggtagctcttgatccggcaaacaaaccaccgctggtagcggtggtttttttgtttgcaagcagcaga

ttacgcgcagaaaaaaaggatctcaagaagatcctttgatcttttctacggggtctgacgctcagtggaacgaaaactca

cgttaagggattttggtcatgagattatcaaaaaggatcttcacctagatccttttaaattaaaaatgaagttttaaatc

aatctaaagtatatatgagtaaacttggtctgacagttaccaatgcttaatcagtgaggcacctatctcagcgatctgtc

tatttcgttcatccatagttgcctgactccccgtcgtgtagataactacgatacgggagggcttaccatctggccccagt

gctgcaatgataccgcgagacccacgctcaccggctccagatttatcagcaataaaccagccagccggaagggccgagcg

cagaagtggtcctgcaactttatccgcctccatccagtctattaattgttgccgggaagctagagtaagtagttcgccag

ttaatagtttgcgcaacgttgttgccattgctacaggcatcgtggtgtcacgctcgtcgtttggtatggcttcattcagc

tccggttcccaacgatcaaggcgagttacatgatcccccatgttgtgcaaaaaagcggttagctccttcggtcctccgat

cgttgtcagaagtaagttggccgcagtgttatcactcatggttatggcagcactgcataattctcttactgtcatgccat

ccgtaagatgcttttctgtgactggtgagtactcaaccaagtcattctgagaatagtgtatgcggcgaccgagttgctct

tgcccggcgtcaatacgggataataccgcgccacatagcagaactttaaaagtgctcatcattggaaaacgttcttcggg

gcgaaaactctcaaggatcttaccgctgttgagatccagttcgatgtaacccactcgtgcacccaactgatcttcagcat

cttttactttcaccagcgtttctgggtgagcaaaaacaggaaggcaaaatgccgcaaaaaagggaataagggcgacacgg

aaatgttgaatactcatactcttcctttttcaatattattgaagcatttatcagggttattgtctcatgagcggatacat

atttgaatgtatttagaaaaataaacaaataggggttccgcgcacatttccccgaaaagtgccacctaaattgtaagcgt

taatattttgttaaaattcgcgttaaatttttgttaaatcagctcattttttaaccaataggccgaaatcggcaaaatcc

cttataaatcaaaagaatagaccgagatagggttgagtgttgttccagtttggaacaagagtccactattaaagaacgtg

gactccaacgtcaaagggcgaaaaaccgtctatcagggcgatggcccactacgtgaaccatcaccctaatcaagtttttt

ggggtcgaggtgccgtaaagcactaaatcggaaccctaaagggagcccccgatttagagcttgacggggaaagccggcga

acgtggcgagaaaggaagggaagaaagcgaaaggagcgggcgctagggcgctggcaagtgtagcggtcacgctgcgcgta

accaccacacccgccgcgcttaatgcgccgctacagggcgcgtcccattcgccattcaggctgcgcaactgttgggaagg

gcgatcggtgcgggcctcttcgctattacgccagtcgaccatagccaattcaatatggcgtatatggactcatgccaatt

caatatggtggatctggacctgtgccaattcaatatggcgtatatggactcgtgccaattcaatatggtggatctggacc

ccagccaattcaatatggcggacttggcaccatgccaattcaatatggcggacttggcactgtgccaactggggaggggt

ctacttggcacggtgccaagtttgaggaggggtcttggccctgtgccaagtccgccatattgaattggcatggtgccaat

aatggcggccatattggctatatgccaggatcaatatataggcaatatccaatatggccctatgccaatatggctattgg

ccaggttcaatactatgtattggccctatgccatatagtattccatatatgggttttcctattgacgtagatagcccctc

ccaatgggcggtcccatataccatatatggggcttcctaataccgcccatagccactcccccattgacgtcaatggtctc

tatatatggtctttcctattgacgtcatatgggcggtcctattgacgtatatggcgcctcccccattgacgtcaattacg

gtaaatggcccgcctggctcaatgcccattgacgtcaataggaccacccaccattgacgtcaatgggatggctcattgcc

cattcatatccgttctcacgccccctattgacgtcaatgacggtaaatggcccacttggcagtacatcaatatctattaa

tagtaacttggcaagtacattactattggaaggacgccagggtacattggcagtactcccattgacgtcaatggcggtaa

atggcccgcgatggctgccaagtacatccccattgacgtcaatggggaggggcaatgacgcaaatgggcgttccattgac

gtaaatgggcggtaggcgtgcctaatgggaggtctatataagcaatgctcgtttagggaac

Legend:

Unhighlighted – pCS2+ backbone

Grey – enzyme cloning site. Note that a BglII site was cloned into a BamHI site upstream of the myristoylation domain

Magenta – Myristoylation domain

Khaki – GS linker

Green – TGFBR1 intracellular domain

Red – TGFBR1 T204D mutation

Turquoise – LOV domain

Yellow – HA tag

Purple – enzyme cloning site, XhoI
