# Supplementary material for: TGF-β uses a novel mode of receptor activation to phosphorylate SMAD1/5 and induce epithelial-to-mesenchymal transition
Source: eLife. 2018 Jan 29;7:e31756. doi: 10.7554/eLife.31756 (PMC5832415; doi:10.7554/eLife.31756)
Supplement: Supplementary file 2. [file elife-31756-supp2.docx]

cgccattctgcctggggacgtcggagcaagcttgatttaggtgacactatagaatacaagctacttgttctttttgcaGGATCCatggggagtagcaagagcaagcctaaggaccccagccagcgcggtggaggaggttctggaggcggtggaagtggtggcggacgaaaatttaaaaggcgcaaccaagaacgcctcaatccccgagacgtggagtatggcactatcgaagggctcatcaccaccaatgttggagacagcactttagcagatttattggatcattcgtgtacatcaggaagtggctctggtcttccttttctggtacaaagaacagtggctcgccagattacactgttggagtgtgtcgggaaaggcaggtatggtgaggtgtggaggggcagctggcaaggggaaaatgttgccgtgaagatcttctcctcccgtgatgagaagtcatggttcagggaaacggaattgtacaacactgtgatgctgaggcatgaaaatatcttaggtttcattgcttcagacatgacatcaagacactccagtacccagctgtggttaattacacattatcatgaaatgggatcgttgtacgactatcttcagcttactactctggatacagttagctgccttcgaatagtgctgtccatagctagtggtcttgcacatttgcacatagagatatttgggacccaagggaaaccagccattgcccatcgagatttaaagagcaaaaatattctggttaagaagaatggacagtgttgcatagcagatttgggcctggcagtcatgcattcccagagcaccaatcagcttgatgtggggaacaatccccgtgtgggcaccaagcgctacatggcccccgaagttctagatgaaaccatccaggtggattgtttcgattcttataaaagggtcgatatttgggcctttggacttgttttgtgggaagtggccaggcggatggtgagcaatggtatagtggaggattacaagccaccgttctacgatgtggttcccaatgacccaagttttgaagatatgaggaaggtagtctgtgtggatcaacaaaggccaaacatacccaacagatggttctcagacccgacattaacctctctggccaagctaatgaaagaatgctggtatcaaaatccatccgcaagactcacagcactgcgtatcaaaaagactttgaccaaaattgataattccctcgacaaattgaaaactgactgtggtggaggaggttctggaggcggtggaagtggtggcggaggtagccctgactacagtctcgtgaaggctctgcaaatggcacaacagaattttgtcattacagacgcctccctcccagacaaccctatcgtctacgccagtagagggtttctgacactgacaggctattctctcgaccagatcctgggcaggaactgcaggtttctgcaagggccagaaacagacccaagagctgtggataagatcaggaatgccatcaccaaaggcgttgataccagtgtctgtctgctgaattatagacaggatggcacaaccttctggaatctcttcttcgtggctggactcagagattctaagggcaatattgtcaactacgtcggagtgcagtcaaaggtgagcgaagattatgccaagctgctggtcaacgagcagaacattgagtacaaaggtgtgcgcaccagtaacatgctgcgcagaaagcccgggtctagttatccgtacgacgtaccagactacgcataaCTCGAGcctctagaactatagtgagtcgtattacgtagatccagacatgataagatacattgatgagtttggacaaaccacaactagaatgcagtgaaaaaaatgctttatttgtgaaatttgtgatgctattgctttatttgtaaccattataagctgcaataaacaagttaacaacaacaattgcattcattttatgtttcaggttcagggggaggtgtgggaggttttttaattcgcggccgcggcgccaatgcattgggcccggtacccagcttttgttccctttagtgagggttaattgcgcgcttggcgtaatcatggtcatagctgtttcctgtgtgaaattgttatccgctcacaattccacacaacatacgagccggaagcataaagtgtaaagcctggggtgcctaatgagtgagctaactcacattaattgcgttgcgctcactgcccgctttccagtcgggaaacctgtcgtgccagctgcattaatgaatcggccaacgcgcggggagaggcggtttgcgtattgggcgctcttccgcttcctcgctcactgactcgctgcgctcggtcgttcggctgcggcgagcggtatcagctcactcaaaggcggtaatacggttatccacagaatcaggggataacgcaggaaagaacatgtgagcaaaaggccagcaaaaggccaggaaccgtaaaaaggccgcgttgctggcgtttttccataggctccgcccccctgacgagcatcacaaaaatcgacgctcaagtcagaggtggcgaaacccgacaggactataaagataccaggcgtttccccctggaagctccctcgtgcgctctcctgttccgaccctgccgcttaccggatacctgtccgcctttctcccttcgggaagcgtggcgctttctcatagctcacgctgtaggtatctcagttcggtgtaggtcgttcgctccaagctgggctgtgtgcacgaaccccccgttcagcccgaccgctgcgccttatccggtaactatcgtcttgagtccaacccggtaagacacgacttatcgccactggcagcagccactggtaacaggattagcagagcgaggtatgtaggcggtgctacagagttcttgaagtggtggcctaactacggctacactagaaggacagtatttggtatctgcgctctgctgaagccagttaccttcggaaaaagagttggtagctcttgatccggcaaacaaaccaccgctggtagcggtggtttttttgtttgcaagcagcagattacgcgcagaaaaaaaggatctcaagaagatcctttgatcttttctacggggtctgacgctcagtggaacgaaaactcacgttaagggattttggtcatgagattatcaaaaaggatcttcacctagatccttttaaattaaaaatgaagttttaaatcaatctaaagtatatatgagtaaacttggtctgacagttaccaatgcttaatcagtgaggcacctatctcagcgatctgtctatttcgttcatccatagttgcctgactccccgtcgtgtagataactacgatacgggagggcttaccatctggccccagtgctgcaatgataccgcgagacccacgctcaccggctccagatttatcagcaataaaccagccagccggaagggccgagcgcagaagtggtcctgcaactttatccgcctccatccagtctattaattgttgccgggaagctagagtaagtagttcgccagttaatagtttgcgcaacgttgttgccattgctacaggcatcgtggtgtcacgctcgtcgtttggtatggcttcattcagctccggttcccaacgatcaaggcgagttacatgatcccccatgttgtgcaaaaaagcggttagctccttcggtcctccgatcgttgtcagaagtaagttggccgcagtgttatcactcatggttatggcagcactgcataattctcttactgtcatgccatccgtaagatgcttttctgtgactggtgagtactcaaccaagtcattctgagaatagtgtatgcggcgaccgagttgctcttgcccggcgtcaatacgggataataccgcgccacatagcagaactttaaaagtgctcatcattggaaaacgttcttcggggcgaaaactctcaaggatcttaccgctgttgagatccagttcgatgtaacccactcgtgcacccaactgatcttcagcatcttttactttcaccagcgtttctgggtgagcaaaaacaggaaggcaaaatgccgcaaaaaagggaataagggcgacacggaaatgttgaatactcatactcttcctttttcaatattattgaagcatttatcagggttattgtctcatgagcggatacatatttgaatgtatttagaaaaataaacaaataggggttccgcgcacatttccccgaaaagtgccacctaaattgtaagcgttaatattttgttaaaattcgcgttaaatttttgttaaatcagctcattttttaaccaataggccgaaatcggcaaaatcccttataaatcaaaagaatagaccgagatagggttgagtgttgttccagtttggaacaagagtccactattaaagaacgtggactccaacgtcaaagggcgaaaaaccgtctatcagggcgatggcccactacgtgaaccatcaccctaatcaagttttttggggtcgaggtgccgtaaagcactaaatcggaaccctaaagggagcccccgatttagagcttgacggggaaagccggcgaacgtggcgagaaaggaagggaagaaagcgaaaggagcgggcgctagggcgctggcaagtgtagcggtcacgctgcgcgtaaccaccacacccgccgcgcttaatgcgccgctacagggcgcgtcccattcgccattcaggctgcgcaactgttgggaagggcgatcggtgcgggcctcttcgctattacgccagtcgaccatagccaattcaatatggcgtatatggactcatgccaattcaatatggtggatctggacctgtgccaattcaatatggcgtatatggactcgtgccaattcaatatggtggatctggaccccagccaattcaatatggcggacttggcaccatgccaattcaatatggcggacttggcactgtgccaactggggaggggtctacttggcacggtgccaagtttgaggaggggtcttggccctgtgccaagtccgccatattgaattggcatggtgccaataatggcggccatattggctatatgccaggatcaatatataggcaatatccaatatggccctatgccaatatggctattggccaggttcaatactatgtattggccctatgccatatagtattccatatatgggttttcctattgacgtagatagcccctcccaatgggcggtcccatataccatatatggggcttcctaataccgcccatagccactcccccattgacgtcaatggtctctatatatggtctttcctattgacgtcatatgggcggtcctattgacgtatatggcgcctcccccattgacgtcaattacggtaaatggcccgcctggctcaatgcccattgacgtcaataggaccacccaccattgacgtcaatgggatggctcattgcccattcatatccgttctcacgccccctattgacgtcaatgacggtaaatggcccacttggcagtacatcaatatctattaatagtaacttggcaagtacattactattggaaggacgccagggtacattggcagtactcccattgacgtcaatggcggtaaatggcccgcgatggctgccaagtacatccccattgacgtcaatggggaggggcaatgacgcaaatgggcgttccattgacgtaaatgggcggtaggcgtgcctaatgggaggtctatataagcaatgctcgtttagggaac

Legend:

Unhighlighted – pCS2+ backbone

Grey – enzyme cloning site, BamHI

Magenta – Myristoylation domain

Khaki – GS linker

Green – ACVR1 intracellular domain

Turquoise – LOV domain

Yellow – HA tag

Purple – enzyme cloning site, XhoI
