# Supplementary material for: TGF-β uses a novel mode of receptor activation to phosphorylate SMAD1/5 and induce epithelial-to-mesenchymal transition
Source: eLife. 2018 Jan 29;7:e31756. doi: 10.7554/eLife.31756 (PMC5832415; doi:10.7554/eLife.31756)
Supplement: Transparent reporting form [file elife-31756-transrepform.docx]

***eLife’s* transparent reporting form**

We encourage authors to provide detailed information *within their submission* to facilitate the interpretation and replication of experiments. Authors can upload supporting documentation to indicate the use of appropriate reporting guidelines for health-related research (see [EQUATOR Network](http://www.equator-network.org/%20)), life science research (see the [BioSharing Information Resource](https://biosharing.org/)), or the [ARRIVE guidelines](http://www.plosbiology.org/article/info:doi/10.1371/journal.pbio.1000412) for reporting work involving animal research. Where applicable, authors should refer to any relevant reporting standards documents in this form.

If you have any questions, please consult our Journal Policies and/or contact us: [editorial@elifesciences.org](mailto:editorial@elifesciences.org).

**Sample-size estimation**

- You should state whether an appropriate sample size was computed when the study was being designed
- You should state the statistical method of sample size computation and any required assumptions
- If no explicit power analysis was used, you should describe how you decided what sample (replicate) size (number) to use

Please outline where this information can be found within the submission (e.g., sections or figure legends), or explain why this information doesn’t apply to your submission:

This does not apply to this work as the paper does not contain any population studies or *in vivo* experiments.

**Replicates**

- You should report how often each experiment was performed
- You should include a definition of biological versus technical replication
- The data obtained should be provided and sufficient information should be provided to indicate the number of independent biological and/or technical replicates
- If you encountered any outliers, you should describe how these were handled
- Criteria for exclusion/inclusion of data should be clearly stated
- High-throughput sequence data should be uploaded before submission, with a private link for reviewers provided (these are available from both GEO and ArrayExpress)

Please outline where this information can be found within the submission (e.g., sections or figure legends), or explain why this information doesn’t apply to your submission:

• As stated in the Methods, experiments were performed at least twice independently (biological duplicates) and the majority were performed at least three times. Within each qPCR experiment, technical triplicates were run.

• RNA-seq experiments (Figure 6 Source data 1) are the means of biological triplicates and the ChIP-seq experiment (Figure 5 Source data 1) is a representative of biological duplicates. Validations of RNA-seq data (Figure 6 – figure supplement 2) and validations of ChIP-seq data (Figure 5C, Figure 5 – figure supplement 1A) are representatives of two biological duplicate experiments, performed in technical triplicate.

• Criteria used for ChIP-seq and RNA-seq data analyses and information regarding the generation of the different datasets presented in the work are described in the Methods.

• The ChIP-seq and RNA-seq data have been submitted to the NCBI Gene Expression Omnibus (GEO) under the accession numbers GSE92443 and GSE103372 (see Methods).

**Statistical reporting**

- Statistical analysis methods should be described and justified
- Raw data should be presented in figures whenever informative to do so (typically when N per group is less than 10)
- For each experiment, you should identify the statistical tests used, exact values of N, definitions of center, methods of multiple test correction, and dispersion and precision measures (e.g., mean, median, SD, SEM, confidence intervals; and, for the major substantive results, a measure of effect size (e.g., Pearson's r, Cohen's d)
- Report exact p-values wherever possible alongside the summary statistics and 95% confidence intervals. These should be reported for all key questions and not only when the p-value is less than 0.05.

Please outline where this information can be found within the submission (e.g., sections or figure legends), or explain why this information doesn’t apply to your submission:

A description of the statistical analysis methods used is given in the Methods.

For each experiment, information on the exact values of N and precision measures can be found in the relevant figure legend.

(For large datasets, or papers with a very large number of statistical tests, you may upload a single table file with tests, Ns, etc., with reference to sections in the manuscript.)

**Group allocation**

- Indicate how samples were allocated into experimental groups (in the case of clinical studies, please specify allocation to treatment method); if randomization was used, please also state if restricted randomization was applied
- Indicate if masking was used during group allocation, data collection and/or data analysis

Please outline where this information can be found within the submission (e.g., sections or figure legends), or explain why this information doesn’t apply to your submission:

This does not apply to this work as the paper does not contain any clinical studies or *in vivo* experiments.

**Additional data files (“source data”)**

- We encourage you to upload relevant additional data files, such as numerical data that are represented as a graph in a figure, or as a summary table
- Where provided, these should be in the most useful format, and they can be uploaded as “Source data” files linked to a main figure or table
- Include model definition files including the full list of parameters used
- Include code used for data analysis (e.g., R, MatLab)
- Avoid stating that data files are “available upon request”

Please indicate the figures or tables for which source data files have been provided:

Source data are as follows:

• **Figure 4 Source data 1.** Source data for ACVR1-IPF fluorescence (panel B)

• **Figure 4 Source data 2.** Source data for ACVR1-IPF fluorescence by flow cytometry (panel C)

• **Figure 5 Source data 1** contains three Excel sheets and is the source data for the ChIP-seq experiment. These data contribute to Figure 5B and Figure 5 – figure supplement 1A.

• **Figure 5 Source data 2.** ChIP-PCR data for graphs in panel C

• **Figure 6 Source data 1** contains three Excel sheets and is the source data for the RNA-seq experiment. The second sheet (Parental vs ΔSMAD1&5) is the source data for the GSEA performed in the third sheet (Relevant pathways).

• **Figure 1 – figure supplement 1 Source data 1.** Source data for qPCRs (panel B)

• **Figure 4 - figure supplement 1 Source data 1.** Source data for ACVR1-IPF fluorescence by flow cytometry (panel E)

• **Figure 4 - figure supplement 1 Source data 2.** Source data for ACVR1-IPF fluorescence (panel F)

• **Figure 4 - figure supplement 1 Source data 3.** Source data for ACVR1-IPF fluorescence by flow cytometry (panel G)

• **Figure 5 – figure supplement 1 Source data 1.** ChIP-PCR data for graphs in panel A

• **Figure 5 – figure supplement 1 Source data 2.** ChIP-PCR data for graphs in panel B

• **Figure 5 - figure supplement 2 Source data 1.** qPCR data for graphs in panel B

• **Figure 5 - figure supplement 2 Source data 2.** qPCR data for graphs in panel C

• **Figure 5 - figure supplement 2 Source data 3.** qPCR data for graphs in panel D

• **Figure 5 - figure supplement 2 Source data 4.** qPCR data for graphs in panel E

• **Figure 6 - figure supplement 2 Source data 1.** qPCR data for all graphs shown.

• **Supplementary file 1** contains the sequence information for the Opto-TGFBR1* construct.

• **Supplementary file 2** contains the sequence information for the Opto-ACVR1 construct.
